# The *BRAF* V600E mutation is a predictor of the effect of radioiodine therapy in papillary thyroid cancer

**DOI:** 10.7150/jca.33105

**Published:** 2020-01-01

**Authors:** Junshang Ge, Jie Wang, Hui Wang, Xianjie Jiang, Qianjin Liao, Qian Gong, Yongzhen Mo, Xiaoling Li, Guiyuan Li, Wei Xiong, Jin Zhao, Zhaoyang Zeng

**Affiliations:** 1 NHC Key Laboratory of Carcinogenesis (Central South University) and Hunan Key Laboratory of Translational Radiation Oncology, Hunan Cancer Hospital and the Affiliated Cancer Hospital of Xiangya School of Medicine, Central South University, Changsha, Hunan, China;; 2 Key Laboratory of Carcinogenesis and Cancer Invasion of the Chinese Ministry of Education, Cancer Research Institute and School of Basic Medicine, Central South University, Changsha, Hunan, China;; 3 Hunan Key Laboratory of Nonresolving Inflammation and Cancer, Disease Genome Research Center, the Third Xiangya Hospital, Central South University, Changsha, Hunan, China.

**Keywords:** I -131 treatment, BRAF V600E, Thyroid function, papillary thyroid carcinoma

## Abstract

**Objective:** To investigate the correlation between the *BRAF* V600E gene mutation and clinicopathological features and thyroid function after iodine-131 treatment in patients with papillary thyroid cancer (PTC).

**Methods**: A total of 128 PTC patients who underwent iodine-131 treatment after a total thyroidectomy from February 2015 to November 2016 at Hunan Cancer Hospital, China, were recruited. There were 25 males and 103 females. The age range was 11 to 73 years old. The *BRAF* V600E mutation in tumor tissues was detected by amplification-restriction mutation system polymerase chain reaction (ARMS-PCR), and the serum levels of Tg, TSH, Tg-Ab, and Tpo-Ab were measured by chemiluminescence after iodine-131 treatment. The *BRAF* V600E mutation was shown to be associated with clinicopathological characteristics and thyroid function indicators after iodine-131 treatment.

**Results**: *BRAF* V600E mutation was detected in 75 of the 128 patients (58.6%) and was observed more frequently in cases with elevated Tg levels (Tg>1.00) at 3, 6, 12, and 18 months after treatment compared with patients without any *BRAF* mutations (*P*<0.05). Patients with *BRAF* V600E mutation had significant lower level of Tg-Ab at 3 and 12 months after treatment with iodine-131 than patients without *BRAF* V600E mutation (*P*<0.05). Among the 75 *BRAF* V600E patients, no significant association was found between the levels of TSH and Tpo-Ab after iodine-131 treatment (*P*>0.05). The *BRAF* V600E mutation was closely associated with the high-risk and age of the patient (≥45 years old) (*P*<0.05), but there was no significant correlation with gender, clinical stage, and distant metastasis.

**Conclusion**: The *BRAF* V600E mutation is closely related to serum Tg elevation after treatment with iodine-131 in papillary thyroid cancer. These findings suggest that this *BRAF* mutation may be a predictor of the efficacy of iodine-131 treatment for papillary thyroid cancer.

## Introduction

Thyroid cancer is the most common malignant tumor of the endocrine system and the most common head and neck cancer. The incidence of thyroid cancer has increased significantly in the past two decades^1^. Thyroid cancer primarily includes the following four pathological types: papillary thyroid carcinoma, follicular thyroid carcinoma, undifferentiated carcinoma and medullary thyroid carcinoma. Among these types, papillary thyroid carcinoma is the most common, accounting for 85% to 90% of the total incidences. Due to the relatively inert biological behavior of PTC, the overall prognosis is relatively good, and the 10-year survival rate of patients is greater than 90%. However, many cases still have a poor prognosis, any many patients eventually die from postoperative recurrence and metastasis. Therefore, predicting the prognosis of papillary thyroid carcinoma and customizing individualized treatment programs have become topics of concern for clinicians.

At present, surgical resection is the preferred treatment for papillary thyroid carcinoma, and this treatment is widely used in clinical practice^2^. After thyroidectomy, adjuvant treatment is usually given, depending on the severity of the condition and the metastasis of the tumor. Among the various adjuvant treatments, I-131 adjuvant radiotherapy is the most common. Thyroid tissue-derived tumor cells usually retain the ability to take up iodine. I-131 treatment can effectively eliminate residual thyroid tissue and micrometastases; therefore, this treatment plays an important role in reducing the recurrence rate and improving survival^3^. Predictive evaluation of the therapeutic effect of I-131 treatment and the development of a corresponding degree of chemotherapy can effectively improve the prognosis of patients with PTC and prolong the recurrence-free survival of patients.

The *BRAF* gene is located on human chromosome 7q34 and encodes a serine/threonine protein kinase with a molecular weight of 67 kD-99 kDs^4^. This protein kinase is involved in regulating the transcriptional activity of cells during cell growth, division and differentiation, and it affects the regulation of cell morphology and the distribution of the cytoskeleton. Approximately 80% of the *BRAF* mutations are an A-T point mutation at the bp 1799, which is known as the *BRAF* V600E mutation. *BRAF* gene mutations are associated with the development of a variety of tumors, such as melanoma^5^, lung cancer^6^, colorectal cancer^7^ and thyroid cancer. In recent years, a large body of literature has shown that the *BRAF* V600E mutation is associated with a series of adverse clinical pathological factors in PTC, such as advanced disease, invasive characteristics, and others^8^-^14^. Many studies have shown that the *BRAF* V600E mutation may result in the activation of downstream genes of the MAPK pathway, which may lead to a slight downregulation of the sodium iodide symporter (NIS), thereby causing an inaccurate NIS localization wherein the NIS cannot be accurately localized to the cell membrane^15^, ^16^. This failure of proper localization leads to a decrease in the ability of tumor cells to take up iodine^17^, ^18^. I-131 adjuvant radiotherapy relies on the uptake of radioactive iodine by tumor cells; therefore, the *BRAF* V600E mutation may lead to a decrease in the therapeutic effect of iodine-131, resulting in a poor prognosis.

The clinical evaluation of the efficacy of I-131 treatment after total thyroid cancer surgery includes imaging examination and tests for thyroid function^19^. The evaluation of the efficacy of I-131 treatment in the thyroid index primarily includes thyroglobulin (Tg), thyroid stimulating hormone (TSH), free T3 (FT3), free T4 (FT4), thyroid peroxidase antibody (TPO-Ab) and thyroglobulin antibody (TG-Ab). Among these factors, dynamic monitoring of thyroglobulin (Tg) levels is important for postoperative follow-up and prognosis evaluation, and such monitoring has been widely used in clinical practice 20, 21. Thyroglobulin glycoprotein is derived from functional thyroid tissue. Under the condition of thyroidectomy, if other physical and chemical conditions are stable, the serum Tg level is directly proportional to the size of thyroid residual tissue.

We primarily investigated the relationship among the *BRAF* V600E gene mutation, the clinicopathological features of PTC patients, and the levels of the thyroid function indicators Tg, TSH, Tg-Ab and Tpo-Ab after iodine-131 treatment.

## Materials and Methods

### Materials

A cohort of 128 patients with PTC who underwent I-131 treatment following total thyroidectomy at Hunan Cancer Hospital from February 2015 to November 2016 were recruited. Patients were eligible for enrolment into the cohort if they had primary papillary thyroid carcinoma and received total thyroidectomy and postoperative iodine-131 treatment. The main exclusion criteria were a diagnosis of double primary cancer and poor physical condition. Written informed consents were obtained from all patients.

There were 25 male and 103 female patients with an age range of 11 to 73 years and a median age of 40 years. According to the International Anti-Cancer Alliance/United States Cancer Society (UICC/AJCC) 2008 staging criteria, 68 cases were stage I, 8 cases were stage II, 8 cases were stage III, and 38 cases were stage IV; the stages of the remaining 6 cases were unknown.

### Methods

Total thyroidectomy and iodine-131 treatment were performed in all cases, and TSH inhibition was performed according to the ATA treatment guidelines. The *BRAF* V600E mutation was detected by amplification-blocking mutation system polymerase chain reaction (ARMS-PCR). The serum levels of Tg, TSH, Tg-Ab and Tpo-Ab were detected by chemiluminescence after the administration of iodine-131 treatment. All testing procedures were performed in compliance with quality control standards.

### Data analysis

The statistical software SPSS for Windows was used for the statistical analyses. The Pearson's chi-square test or a t-test were used to analyze the relationships among the presence of the *BRAF* mutation, the clinicopathological features of patients and the results of the thyroid function tests. When *P*<0.05, differences were considered statistically significant.

## Results

### Relationship between the *BRAF*V600E mutation and clinicopathological features

Among all the patients, 75 cases were positive for the *BRAF* V600E mutation, and 53 patients carried the wild type *BRAF* gene, thus the mutation rate was 58.6%. Among the patients with the *BRAF* V600E mutation, there were 17 males and 58 females; among the patients with wild-type *BRAF*, there were 8 males and 47 females (*P*=0.25). The median age of patients with the *BRAF* mutation was 41 years, while the median age of patients with wild-type *BRAF* was 38 years (*P*=0.12). The *BRAF* V600E mutation was closely associated with the high-risk age (≥45 years) (*P*=0.025). There was no significant correlation between the* BRAF* V600E mutation and lymph node metastasis (*P*=0.059), distant metastasis (*P*=0.76), or clinical stage (*P*=0.26) (Table 1).

### *BRAF*V600E mutation and thyroid function following iodine-131 treatment

**Figure 1:**
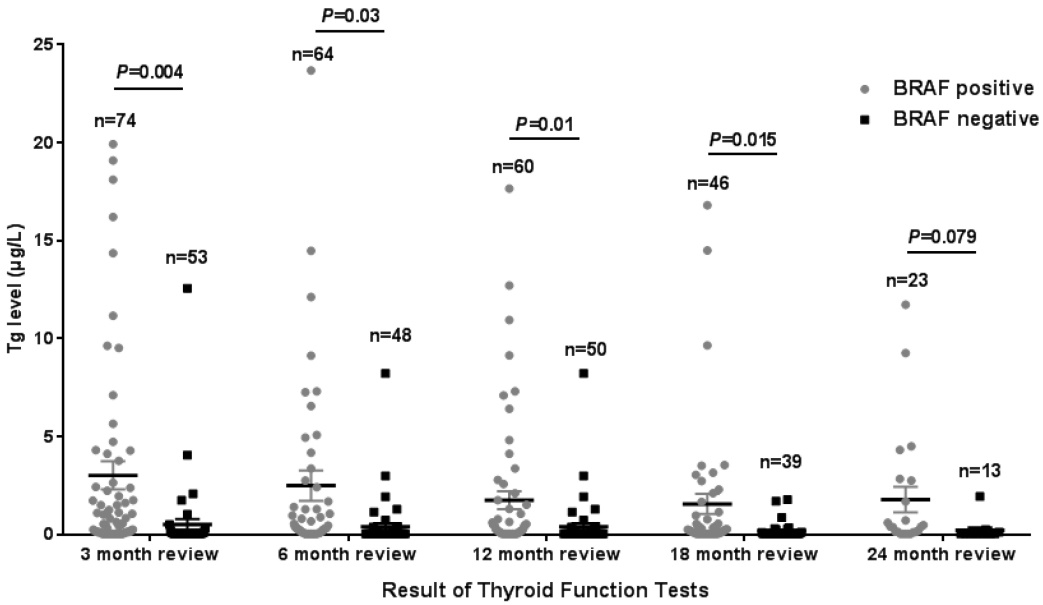
Tg levels in the 3rd, 6th, 12th, 18th, and 24th months after thyroid cancer treatment with iodine-131 in 128 patients from Hunan Cancer Hospital (error bar: mean with SEM).

**Figure 2:**
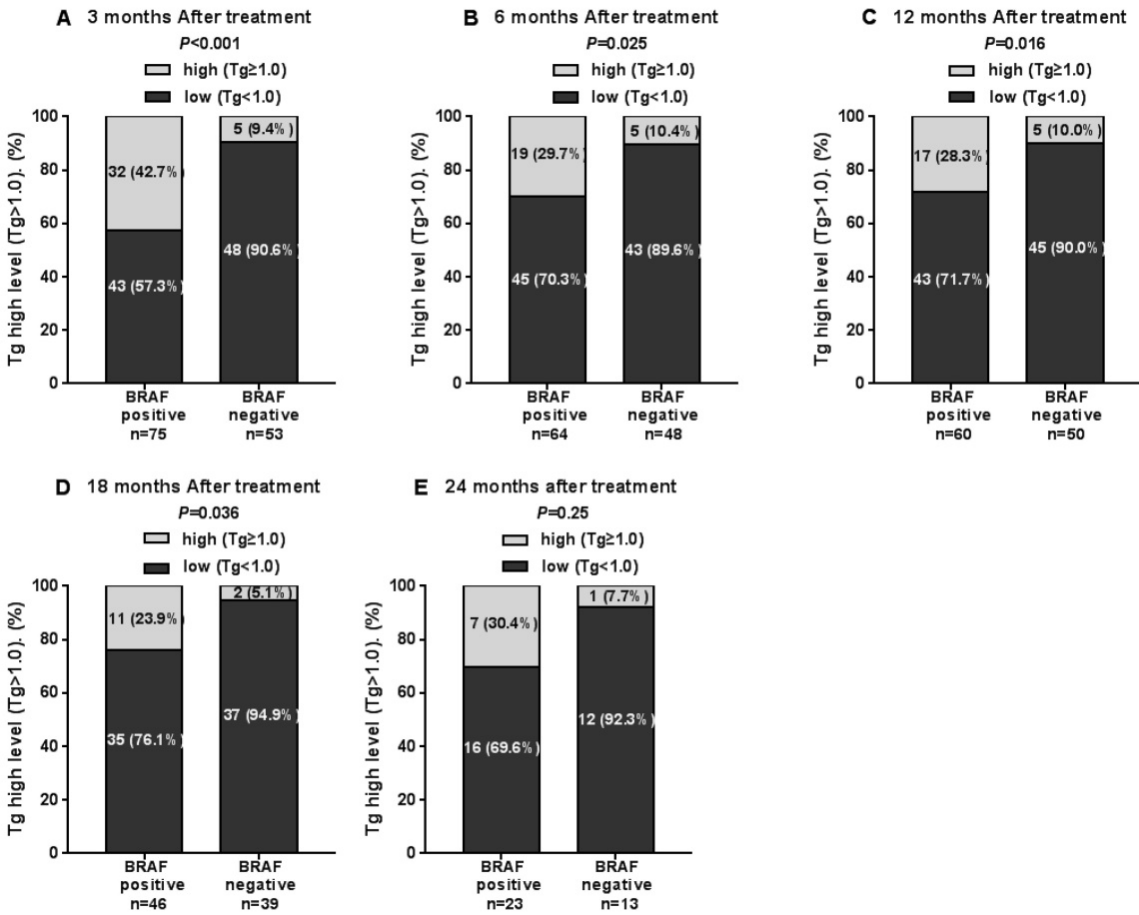
The rate of elevated Tg levels in the 3rd, 6th, 12th, 18th and 24th months after thyroid cancer treatment with iodine-131.

**Figure 3:**
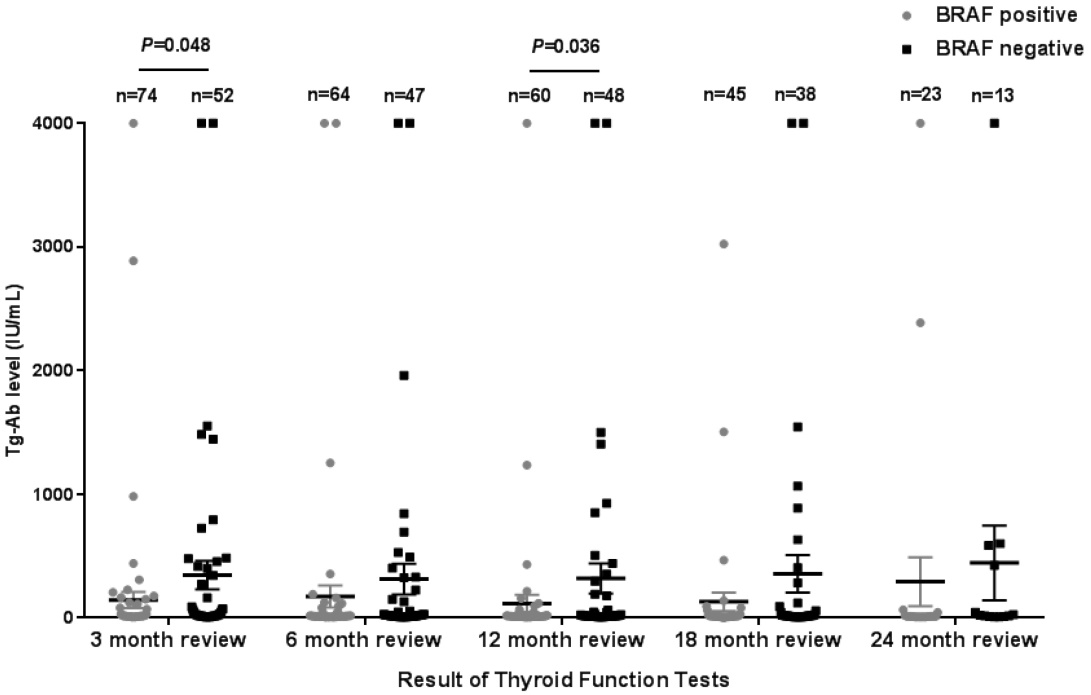
Tg-Ab levels in the 3rd (A), 6th (B), 12th (C)and 18th (D) month after thyroid cancer treatment with iodine-131in 128 patients from Hunan Cancer Hospital (error bar: mean with SEM).

The Tg levels at 3, 6, 12, and 18 months after iodine-131 treatment in patients with the *BRAF* mutation were significantly higher compared with those in patients with wild-type *BRAF* (*P*<0.05) (Table 2, Fig. 1). *BRAF* V600E-mutation-patients compared with *BRAF* V600E-WT-patients, under 3 months treatment, the mean Tg level was 3.02 μg/L vs 0.54 μg/L, and the median Tg level was 0.54 μg/L vs 0.04 μg/L (*P*=0.004); at 6 months after treatment, the mean Tg level was 2.50 μg/L vs 0.51 μg/L, and the median Tg level was 0.30 μg/L vs 0.04 μg/L (*P*=0.03); at 12 months after treatment, the mean Tg level was 3.65 μg/L vs 0.39 μg/L, and the median Tg level was 0.26 μg/L vs 0.04 μg/L (P=0.010); and at 18 months after treatment, the mean Tg level was 1.74 μg/L vs 0.18 μg/L, and the median Tg level was 0.28 μg/L vs 0.04 μg/L (*P*=0.015). The rate of elevated Tg levels (Tg>1.00) at 3, 6, 12, and 18 months after treatment was significantly higher in patients with the *BRAF* mutation than in patients with wild-type *BRAF* (44.5% vs 15.2%, 29.7% vs 10.4%, 27.8% vs 11.1%, 27.6% vs 5.1%) (*P*<0.05) (Table 2, Fig. 2). The level of Tg-Ab at 3 and 12 months after treatment with iodine-131 in patients with the *BRAF* mutation was significantly lower than in patients with wild-type *BRAF* (*P*<0.05) (Table 2, Fig. 3). The mean level of Tg-Ab was 94.10 IU/mL vs 274.85 IU/mL, and the median Tg-Ab level was 16.31 IU/mL vs 26.93 IU/mL (*P*=0.048). At 12 months after treatment, the mean Tg-Ab level was 52.74 IU/mL vs 241.31. IU/mL, and the median Tg-Ab level was 13.47 IU/mL vs 17.46 IU/mL (*P*=0.036). Due to the tracking time limit, there were only 36 cases that could be traced to 24 months after treatment, including 23 cases of *BRAF*-positive patients and 13 cases of patients with wild-type *BRAF*. No significant differences were detected between *BRAF* mutational status and thyroid function parameters, which may be due to the low number of cases (Tg mean=1.78 μg/L vs 0.21 μg/L, median=0.40 μg/L vs 0.04 μg/L, *P*=0.079). There was no correlation between *BRAF* V600E mutational status and the levels of TSH and Tpo-Ab after iodine 131-treatment (*P*>0.05) (Table 2).

### Clinicopathological features and thyroid function following iodine-131 treatment

Based on our sample size and follow-up time, there was no correlation between thyroid function and patient's age, gender, clinical stage, lymphovascular invasion, and metastasis (*P*>0.05).

## Discussion

Thyroid cancer is a common endocrine tumor with an increasing prevalence in China year by year^22^-^24^. The four primary histological types include papillary carcinoma, medullary carcinoma, undifferentiated carcinoma, and follicular carcinoma 25, among which papillary thyroid cancer (PTC) is the most common, accounting for 85% to 90% of the total incidence. The clinical biological characteristics of thyroid cancer with different pathological types are also different. Papillary thyroid carcinoma is one of the less malignant thyroid cancers, which shows low lymph node metastasis rate and distant metastasis rate. Clinically, in addition to the general clinical features, most papillary thyroid carcinoma patients have considerable variable clinical manifestations. This situation may be caused by many factors, including age, gender, living environment, genetic background, and gene mutation.

In recent years, with advances in gene sequencing technology, molecular diagnosis has been widely used for the early diagnosis of cancer, the determination of radiotherapy and chemotherapy, and the postoperative management of patients. In the molecular diagnosis of thyroid cancer, the related molecular markers include TERT, *BRAF*, PAX8/ PPARγ, RAS and RET/PTC^26^. The *BRAF* V600E gene mutation is one of the most common mutations in PTC^27^, ^28^ and is closely related to the occurrence, development, biological behavior and prognosis of PTC^29^, ^30^. Studies have shown that an assessment of the *BRAF* V600E mutational status can be combined with cell morphological analysis to aid in the diagnosis of PTC in cytologically uncertain thyroid nodules^31^. Therefore, we selected 128 patients with PTC who underwent iodine-131 treatment after total thyroidectomy in the Cancer Hospital of Hunan Province from February 2015 to November 2016. We investigated the relationships among the *BRAF* V600E mutation, clinical pathological features and the efficacy of iodine-131 treatment. Among the 128 patients with PTC included in this study, the *BRAF* V600E mutation rate was 58.6%. In a previous meta-analysis of *BRAF* V600E mutation in 25241 cases with PTC, 15290 (60.6%) were positive for *BRAF* mutation and 9,951 (39.4%) were tested negative for *BRAF* mutation^32^. The inclusion and exclusion criteria for that meta-analysis are basically similar to ours. And the *BRAF* V600E mutation rate was also similar to our finding.

In this study, we compared the clinical characteristics between patients with the *BRAF* mutation and patients with wild-type *BRAF* and found that the *BRAF* mutation was significantly more frequent in patients of the high-risk age (>45 years old). In the current prognosis evaluation system of thyroid cancer, the patient's age is a recognized risk factor for death; various clinical guidelines and risk assessment models include the patient's age as the primary risk factor in the management and prognostic evaluation of thyroid cancer. The presence of the *BRAF* mutation is closely related to the patient's age; thus, there may be synergistic effects leading to a poor prognosis^33^. Therefore, in the risk assessment of PTC patients, the *BRAF* gene mutation and the patient age should be considered as factors associated with a poor prognosis. This finding has a certain reference value for the diagnosis and treatment of patients of a high-risk age (>45 years old). However, due to the small sample size and short follow-up time, there was no significant correlation between the *BRAF* mutation and gender, lymphatic vessel invasion, distant metastasis or clinical stage in this study.

At present, there are few studies on the correlation between *BRAF* mutational status and iodine-131 treatment, and the results are controversial. According to the guidelines, the current strategies for the treatment of thyroid cancer are primarily surgical treatment, radioactive iodine treatment and TSH inhibition treatment; molecular-targeted therapy has gradually entered the field of vision as an emerging treatment program^34^. The clinical evaluation of the efficacy of iodine-131 treatment typically includes imaging and tests for thyroid function^35^. The key indicators of thyroid function are the levels of thyroglobulin (Tg), thyroid stimulating hormone (TSH), and thyroglobulin antibody (Tg-Ab)^36^. We focused on the relationship between gonadotropin function and *BRAF* V600E mutational status after iodine-131 treatment.

Patients who were positive for the *BRAF* V600E mutation had significantly higher Tg levels compared with patients with wild-type *BRAF*. Tg is a glycoprotein that is derived from functional thyroid tissue, accounting for 75% of all thyroid proteins. Tg is a macromolecular glycoprotein secreted by thyroid follicular epithelial cells and consists of two identical monomeric peptide chains with a molecular weight of 660 kDa^37^. Under normal circumstances, Tg circulates within thyroid cells, and only a very small portion of the Tg is secreted into the blood through the thyroid lymphatic vessels. Under normal conditions, the residual tissue size of the thyroid is proportional to the serum levels of Tg. Therefore, after iodine-131 treatment following PTC total resection, the serum levels of Tg directly reflect the size of the residual thyroid tissue and micrometastases, and an increase in these levels indicate an increase in the residual thyroid tissue and micrometastases^38^. The Tg level of patients who were positive for the *BRAF* V600E mutation was significantly increased, suggesting that the therapeutic effect and prognosis of iodine-131 treatment are relatively poor in these patients. Other studies suggest that Tg level is a key predictor of iodine-131 treatment and PTC prognosis. After iodine-131 treatment following total thyroidectomy, the serum level of thyroglobulin was less than 1 μg/L, which is used as the standard for radioactive iodine ablation in patients with low-risk thyroid papillary carcinoma^39^. A Tg level higher than 1 μg/L indicates that the treatment effect of iodine-131 is not good. The rate of elevated Tg levels (>1.0 μg/L) was significantly increased in patients who were positive for the *BRAF* mutation (*P*<0.05). This finding indicates that the treatment effect of iodine-131 was worse in these patients than in patients with wild-type *BRAF*. Recent studies indicate that the *BRAF* V600E mutation can lead to a downregulation of iodine metabolism because the *BRAF* V600E mutation leads to the continuous activation of the downstream genes of the MAPK pathway, resulting in the decreased expression and abnormal localization of the sodium iodide symporter (NIS). In the case of wild-type *BRAF,* NIS is primarily located in the thyroid follicular membrane; however, under conditions of the *BRAF* V600E mutation, NIS cannot be accurately localized to the follicular membrane and is more dispersed in the cytoplasm^40^. Therefore, patients with the *BRAF* V600E mutation exhibit poor iodine absorption during iodine-131 treatment, which renders the treatment less effective^41^, leading to a poor prognosis. The findings of this study suggest that *BRAF* mutation can be used as a predictor of the efficacy of iodine-131 treatment.

Tg-Ab is a common autoantibody in the serum of patients with autoimmune thyroid disease. This antibody is primarily composed of IgG1, IgG2 and IgG4, and a small portion is IgA and IgM. TG-Ab is generally considered to cause no damage to the thyroid gland. At 3 months and 12 months after iodine-131 treatment, the Tg-Ab levels in patients with the *BRAF* mutation were significantly lower than in patients with wild-type *BRAF*. Regarding the role of Tg-Ab in the development of PTC, current research is sparse and contradictory. According to Rakib et al., the trend of postoperative Tg-Ab elevation can be used as a rough estimate of prognosis^42^, ^43^. According to Velsen et al., the continued increase in Tg-Ab may suggest tumor recurrence^44^. Smooke-Praw et al. conclude that the level of Tg-Ab cannot predict the disease state of PTC^45^, whereas Wu et al. state that the serum levels of Tg-Ab can be used as an independent predictor of PTC^46^. The results of the present study suggest that the *BRAF* mutation may reduce the levels of Tg-Ab in the serum, but the specific mechanism warrants further exploration.

Studies have suggested that patients with the *BRAF* V600E mutation should be overtreated to some extent^47^, because *BRAF* mutations are an independent risk factor for lymph node metastasis and are closely related to tumor size and invasiveness^48^-^57^. Both the clinical studies and the basic medical studies performed here suggest that the presence of the *BRAF* mutation may lead to a decrease in the efficacy of iodine-131 treatment. Therefore, we suggest that a higher level of radioactive iodine-131 treatment should be considered for patients who are positive for this mutation.

In recent years, individualized treatment has gradually gained attention, and the individualized treatment of cancer has been increasingly promoted^58^-^63^. As the gene with the highest mutation rate in PTC, the *BRAF* gene is of great significance for the development of PTC and is widely used in clinical practice. Testing for the *BRAF* V600E mutation is important, as this mutation is involved in the following: early diagnosis of thyroid cancer with FNC, independent risk factor prediction of PTC high-risk age, independent risk factor prediction of PTC lymph node vascular invasion and lymph node metastasis, prognosis evaluation of PTC, and posttreatment management^64^-^70^. The results of this study suggest that *BRAF* mutations should be used as an important indicator when developing individualized treatment options for patients with PTC. According to the results of this study, the *BRAF* V600E gene mutation is closely related to serum Tg elevation after PTC iodine-131 treatment, suggesting that this mutation may be used as a predictor of the efficacy of PTC I-131 treatment.

## Figures and Tables

**Table 1:** Comparison of *BRAF* V600E mutation status and clinicopathological features of 128 patients with PTC.

Variables	No. of patients	*BRAF* mutation		*P* value
		Positive	Negative	
**Age, year**				
<45	82	42	40	0.025
≥45	46	33	13	
**Gender**				
Male	25	17	8	0.25
Female	103	58	47	
**Clinical stage**				
I	68	38	30	0.26
II	8	3	5	
III	8	5	3	
IV	38	26	12	
NA	6	3	3	
I+ III	76	41	35	0.14
III+IV	46	31	15	
**Lymphovascular Invasion**				
No	7	2	5	0.059
Yes	121	73	48	
**Metastasis**				
No	117	69	48	0.76
Yes	11	6	5	

**Table 2:** Correlation between *BRAF* mutation status and thyroid function in 128 patients with papillary thyroid carcinoma.

Test items	3 months review		*P* value	6 months review		*P* value	12 months review		*P* value	18 months review		*P* value	24 months review		*P* value
	*BRAF*+	*BRAF*-		*BRAF*+	*BRAF*-		*BRAF*+	*BRAF*-		*BRAF*+	*BRAF*-		*BRAF*+	*BRAF*-	
Tg Average (μg/L)	3.02	0.54	0.004	2.50	0.51	0.03	3.65	0.39	0.010	1.74	0.18	0.015	1.78	0.21	0.079
Tg Median (μg/L)	0.54	0.04		0.30	0.04		0.26	0.04		0.28	0.04		0.40	0.04	
Rate of elevated Tg (≥1.00)	42.7%	9.4%	<0.001	29.7%	10.4%	0.025	28.3%	10.0%	0.016	27.6%	5.1%	0.036	30.4%	7.7%	0.25
TSH Average (IU/mL)	0.930	0.780	0.45	0.370	0.290	0.68	1.098	1.555	0.65	0.222	0.281	0.60	0.398	0.107	0.60
TSH Median (IU/mL)	0.114	0.082		0.072	0.036		0.066	0.082		0.0545	0.0505		0.101	0.042	
Tg-Ab Average (IU/mL)	94.10	274.85	0.048	115.05	233.95	0.29	52.74	241.31	0.036	131.49	257.77	0.09	293.31	445.07	0.66
Tg-Ab Median (IU/mL)	16.31	26.93		14.68	22.05		13.47	17.46		14.08	18.99		14.15	21.85	

## Abbreviations

PTC: Papillary Thyroid Cancer
Tg: thyroglobulin
TSH: thyroid stimulating hormone
Tg-Ab: thyroglobulin antibody
Tpo-Ab: thyroid peroxidase antibody
NIS: sodium iodide symporter
ARMS-PCR: amplification-blocking mutation system polymerase chain reaction
